# Case report: Successful rescue of an adult patient with diquat poisoning complicated by rhabdomyolysis and compartment syndrome

**DOI:** 10.1186/s12245-026-01254-6

**Published:** 2026-06-08

**Authors:** Yonghui Zhang, Xisheng Zheng, Zhang Tai’an, Feng Yongli, Zhang Chaoyuan, Sun Xiuqin, Jia Mingya, Li Changli, Xiao Jing, Ma Jing, Zhou Xiaochao, Wei Chao, Bai Bin

**Affiliations:** 1 Department of Surgery Critical Care Medicine, Fuwai Hospital, Chinese Academy of Medical Sciences and Beijing Union Medical School, Beijing, China; 2 Department of Critical Care Medicine, Nanyang Central Hospital, Nanyang, Henan, 473000 China; 3 Department of Critical Care Medicine, Dengzhou People’s Hospital, Dengzhou, Henan, 474150 China

**Keywords:** Diquat poisoning, Rhabdomyolysis, Osteofascial compartment syndrome

## Abstract

**Objective:**

To summarize the clinical experience of successfully treating a patient with diquat poisoning complicated by rhabdomyolysis and compartment syndrome at Nanyang Central Hospital, and to provide references for the diagnosis, treatment, and early identification of complications of such severe poisoning.

**Methods:**

The clinical data of 1 patient with diquat poisoning combined with multiple complications were retrospectively analyzed. After the patient took diquat voluntarily, he first received basic treatments such as gastric lavage and catharsis at a local hospital, and was transferred to our hospital due to critical condition. Relevant examinations were completed after admission, and the patient was diagnosed with diquat poisoning, acute kidney injury, and rhabdomyolysis; compartment syndrome was identified in a timely manner subsequently. The treatment included hemoperfusion combined with hemofiltration, decompressive fasciotomy, and symptomatic supportive treatment throughout the course.

**Results:**

After comprehensive treatment, the patient’s poisoning symptoms were controlled, the indicators related to acute kidney injury and rhabdomyolysis returned to normal, compartment syndrome was effectively relieved, limb function was preserved, and the patient was finally cured and discharged without serious sequelae.

**Conclusion:**

Cases of diquat poisoning complicated by rhabdomyolysis and compartment syndrome are rare and life-threatening. Early definite diagnosis, timely application of blood purification therapy to remove toxins, and vigilance and early intervention of complications (such as decompressive fasciotomy for compartment syndrome) are the keys to improving the success rate of treatment and achieving limb salvage. Clinicians should attach great importance to the comprehensive treatment and complication monitoring of such severe poisoning.

## Introduction

Diquat (1,1’-ethylene-2,2’-bipyridilium) is a nonselective bipyridyl herbicide, related structurally to paraquat [1]. The national poison data system of the United States revealed 2,128 cases of diquat poisoning between 1998 and 2013 [2]. Diquat, like paraquat, is a potent redox cycler and its toxic effects depend on its ability to undergo a single electron addition to form a free radical. This occurs in the presence of NADPH (nicotinamide adenine dinucleotide phosphate) and cytochrome P450 reductase [3]. The diquat radical formed in this step is highly unstable and transfers an electron to molecular oxygen to form a superoxide anion radical, a highly reactive species. The superoxide anion radicals produced from the redox cycling of diquat react with each other forming hydrogen peroxide and molecular oxygen. In the presence of iron, the superoxide anion radical reacts with hydrogen peroxide generating the even more potent hydroxyl radical. The hydroxyl radical can attack the lipid chains of biological membranes initiating lipid peroxidation which causes membrane damage and ultimately cell death [4]. Acute diquat poisoning damaged the kidney, liver, and central nervous systems, and subsequent multiple-organ failure syndromes were the main cause of death [5]. Diquat poisoning caused acute kidney injury (AKI) as much as 73.3%, which was higher than other types of pesticide poisoning [6–8]. In cases of severe diquat poisoning, complications such as respiratory failure, heart failure, arrhythmia, epilepsy, and even intracerebral hemorrhage may be induced [9–10]. Rhabdomyolysis is rarely reported in diquat poisoning [11–12]. The first reported patient with diquat poisoning-induced compartment syndrome eventually failed to be rescued and died of respiratory failure [13].

## Case description

**Fig. 1:**
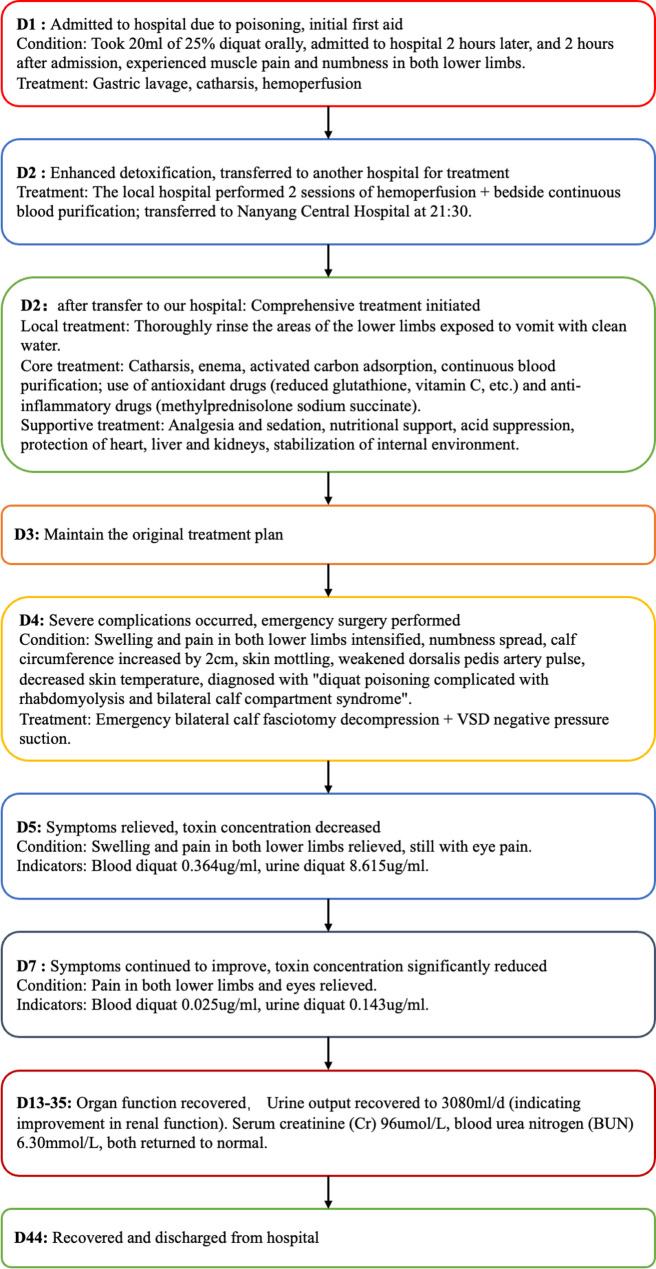
The treatment flowchart for the patient

Patient: This flowchart outlines the patient’s complete therapeutic course(Fig. 1).Male, 22 years old, admitted to our hospital with the main complaint of “sore throat for more than 1 day after oral ingestion of ‘diquat’”. The patient took 20 ml of 25% diquat orally one day ago, and a small amount of diquat was vomited onto his lower limbs, followed by sore throat. He went to the emergency department of the local county hospital 2 hours later, and anuria occurred there. A complete blood count was performed: WBC12.81 × 10^9/L, N84.7%, CRP 0.16 mg/L; Blood biochemistry: UA533umol/L, CK 99U/L (reference range: 39-308U/L), Cr 76 umol/L(reference range: 26–140 U/L), BUN6.09mmol/L (reference rage༚3.2–7.1 mmol/L), Toxicant monitoring showed: blood diquat concentration: 78.571ug/ml, urine diquat concentration: 0.472ug/ml. Acute pesticide poisoning, liver function damage, and renal failure were considered. After admission, treatments such as gastric lavage, fluid replacement, two sessions of HA330 hemoperfusion, and Hemoperfusion and Hemofiltration were immediately given. Two hours after admission to the local hospital, the patient developed bilateral lower limb muscle pain and numbness.

Day 2: Re-examination showed significant abnormalities: white blood cell count 16.7 × 10^9/L (neutrophil ratio 87.6%), creatine kinase (CK) 5680.9 U/L, myoglobin > 1200 ng/ml (reference range: 0–70 ng/ml), and serum creatinine (Cr) 186.2 µmol/L, suggesting rhabdomyolysis and progressive acute kidney injury. Due to the critical condition, the patient was transferred to the ICU of our hospital.

On admission examination: T 36.6℃, P 78 beat/min, R 17 breath/min, BP 101 /62mmHg, H 175 cm, W 70.0 kg, BMI22.9(kg/m^2^), SPO_2_༈air༉100%, The patient was conscious and pushed into the ward. Physical examination: pharynx was red with scattered ulcers, and the patient complained of eye pain; no other abnormalities were found. Test report of lymphocyte immune analysis (relative count + absolute count): Percentage of helper/inducer T lymphocytes: 29.38% (↓),Absolute count of lymphocytes: 322.00 cells/µl (↓),Absolute count of total T lymphocytes: 183.00 cells/µl (↓),Absolute count of helper/inducer T lymphocytes: 94.00 cells/µl (↓),Absolute count of suppressor/cytotoxic T lymphocytes: 84.00 cells/µl (↓),Absolute count of B lymphocytes: 71.00 cells/µl (↓),Absolute count of NK lymphocytes: 67.00 cells/µl (↓)Immediately, the local areas of the lower limbs exposed to vomited diquat were thoroughly rinsed with clean water. Treatments including catharsis, enema, activated charcoal adsorption of toxins, fluid replacement, urine alkalinization, and blood purification were administered. Antioxidant and free radical scavenging therapies with reduced glutathione, vitamin C, and acetylcysteine, as well as anti-inflammatory treatment with methylprednisolone sodium succinate were given. Meanwhile, analgesia and sedation, nutritional support, acid suppression to prevent gastrointestinal bleeding, myocardial nutrition, liver function protection, renal support, and stabilization of the internal environment were provided.

**Fig. 2:**
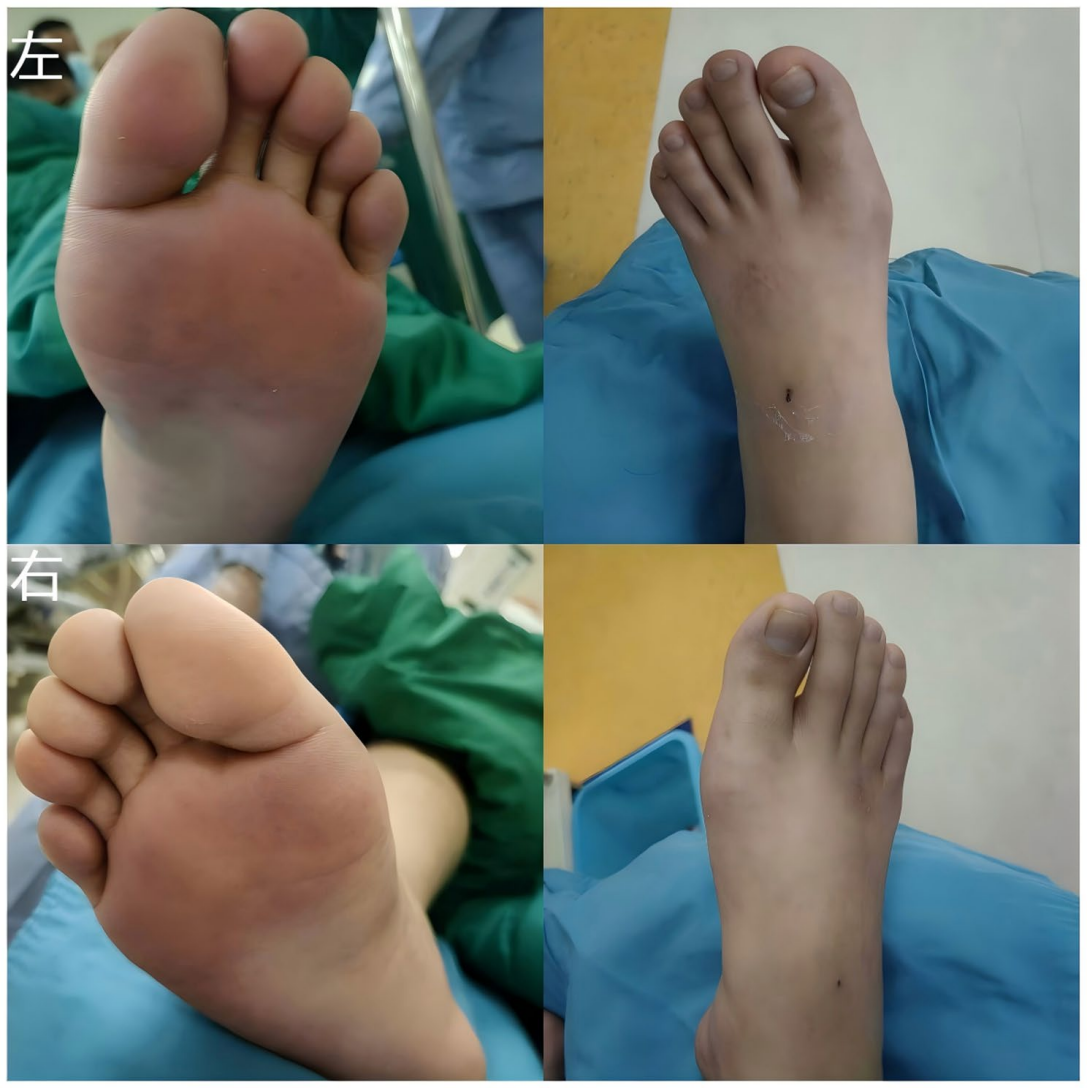
The skin of the patient’s lower extremities showed alternating red and white mottling

Day 4: the patient’s bilateral lower limb swelling and pain aggravated, accompanied by numbness below the mid-shaft of the lower legs. The circumference of both lower legs increased by 2 cm compared with the previous day. Color Doppler ultrasound excluded lower limb arterial and venous thrombosis; physical examination showed mottled red-white skin of the lower limbs, weakened bilateral dorsalis pedis artery pulses, and decreased skin temperature of the feet and toes—consistent with the clinical manifestations of compartment syndrome (paresthesia, pallor, pulselessness). (Fig. 2).

**Fig. 3:**
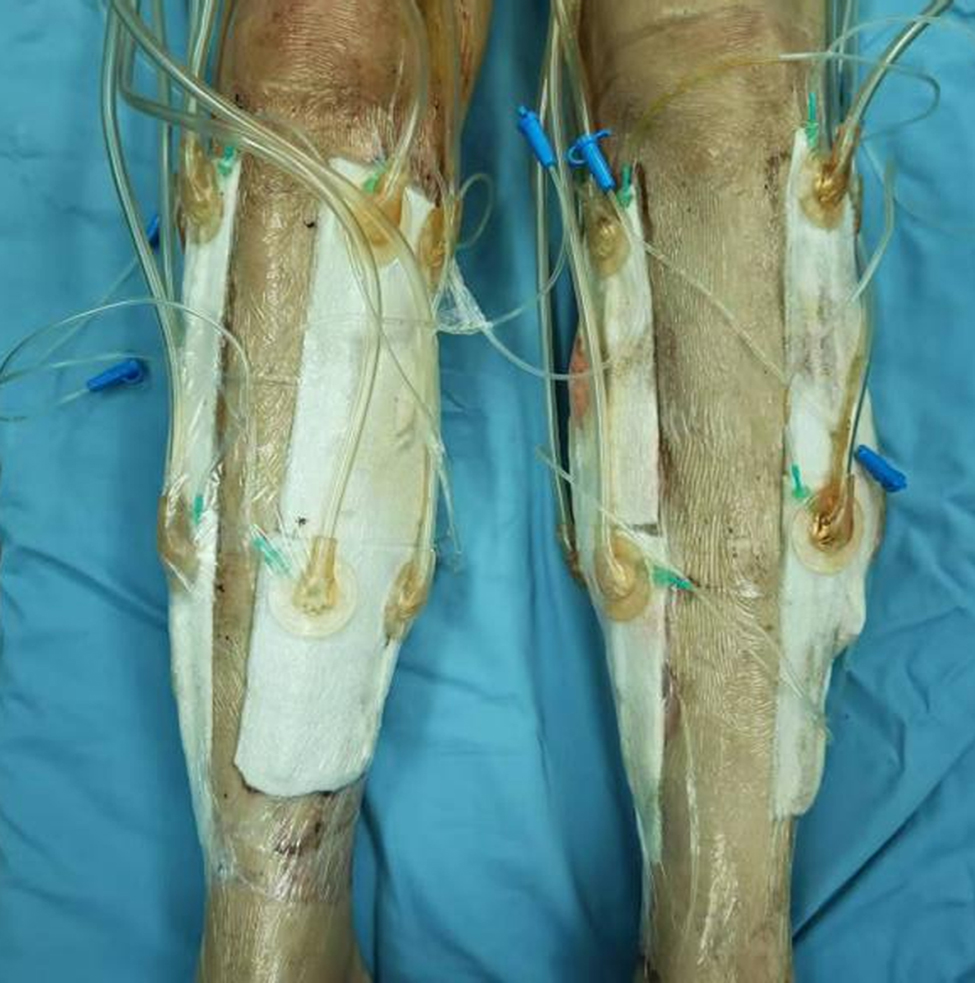
Emergency fasciotomy for decompression with VSD (Vacuum Sealing Drainage) negative pressure suction was performed on both lower extremities

It was considered as diquat poisoning complicated with rhabdomyolysis and bilateral lower leg compartment syndrome. Emergency bilateral lower leg fasciotomy for decompression with VSD (vacuum sealing drainage) negative pressure suction was performed (Fig. 3).

On the 5th day, the distending pain in the lower limbs was relieved, but eye pain still persisted. Re-examination showed that the blood diquat concentration was 0.364ug/ml, and the urine diquat concentration was 8.615ug/ml.

On the 7th day, the pain in both lower limbs and eyes was relieved. Re-examination showed that the blood diquat concentration was 0.025 µg/ml, and the urine diquat concentration was 0.143 µg/ml. The re-examined blood diquat concentrations during the patient’s hospitalization are shown in Table 1 below:

**Table 1:** Diquat concentrations in blood and urine

Time	D1	D3	D5	D7	D10
Blood diquat concentration(ug/ml)	78.571	2.573	0.364	0.025	0.015
Urine diquat concentration(ug/ml)	0.472	4.308	8.615	0.143	0.756

**Fig. 4:**
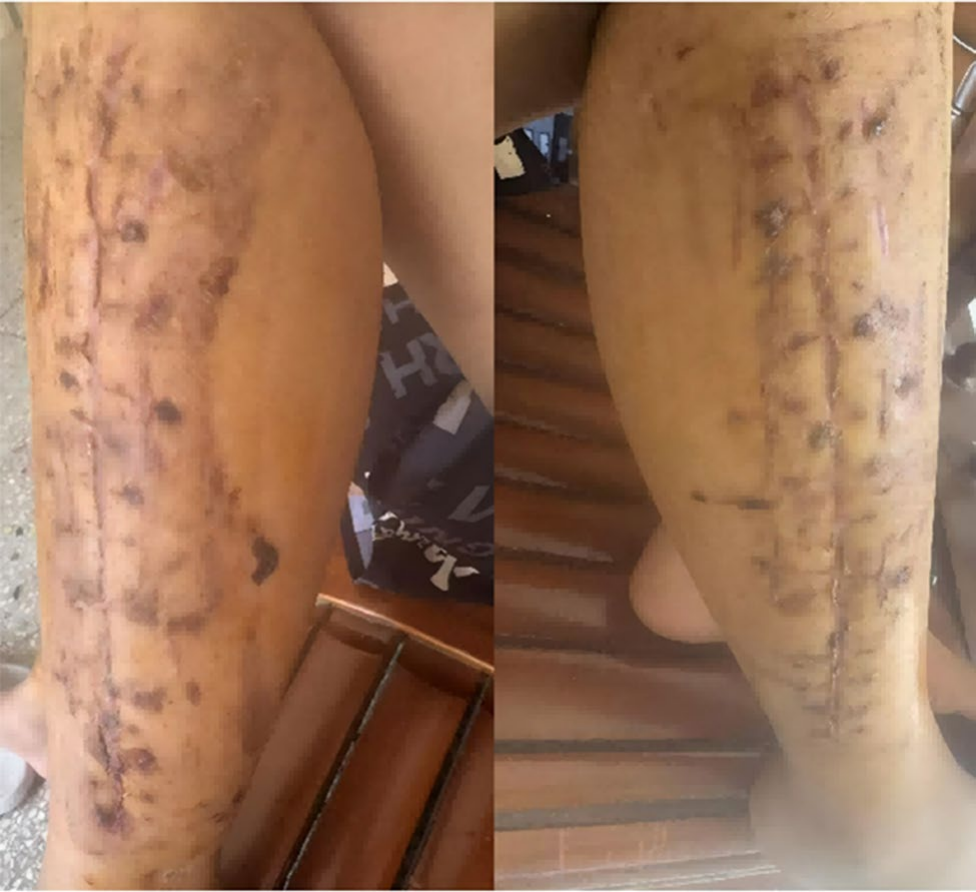
Follow-up after discharge showed that the wounds on the patient’s lower extremities healed well

On the 13th day, the patient’s urine output was 3080 ml/day. On the 35th day after the onset, the blood creatinine (Cr) was 96 µmol/L and blood urea nitrogen (BUN) was 6.30 mmol/L, both of which returned to normal. Subsequently, the incisions on both lower limbs healed, and the patient was discharged. The patient’s wound healed well and they were discharged smoothly after the sutures were removed. The patient’s wound healed well, and the sutures were removed. Following an uneventful recovery in hospital, the patient was discharged. Due to the extended length of the hospitalization, the patient elected to transfer to a local facility for subsequent rehabilitation.The patient was followed up 10 days after discharge, and he recovered well with only a slight limp in the left lower limb. At the two-month follow-up after discharge, the patient exhibited complete resolution of symptoms in the lower extremities (Fig. 4).

**Fig. 5:**
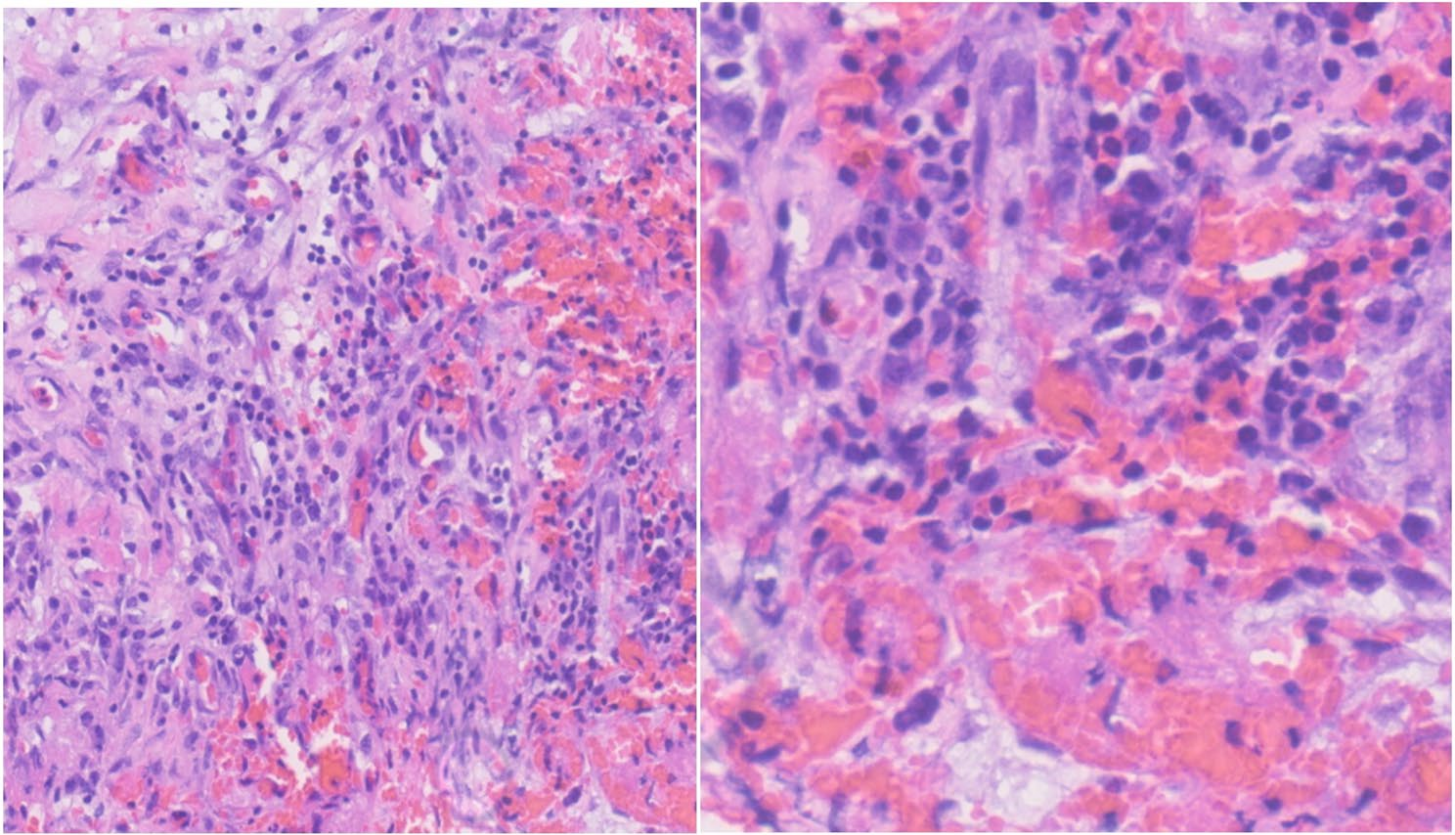
Lower extremity muscle biopsy: (Calf muscle) Lymphocytes and scattered eosinophils were observed between muscle tissues; hemorrhage, inflammatory necrosis and suppurative inflammation were present in some areas; in addition, irregular, structureless substances with light red staining (considered as gelatin sponge) were seen

In the pathological analysis of the skeletal muscle of the affected limb in this case, lymphocytes and scattered eosinophils were found in the muscle tissue(Fig. 5), which provides more active schemes and ideas for further precise treatment of diquat poisoning.

**Fig. 6:**
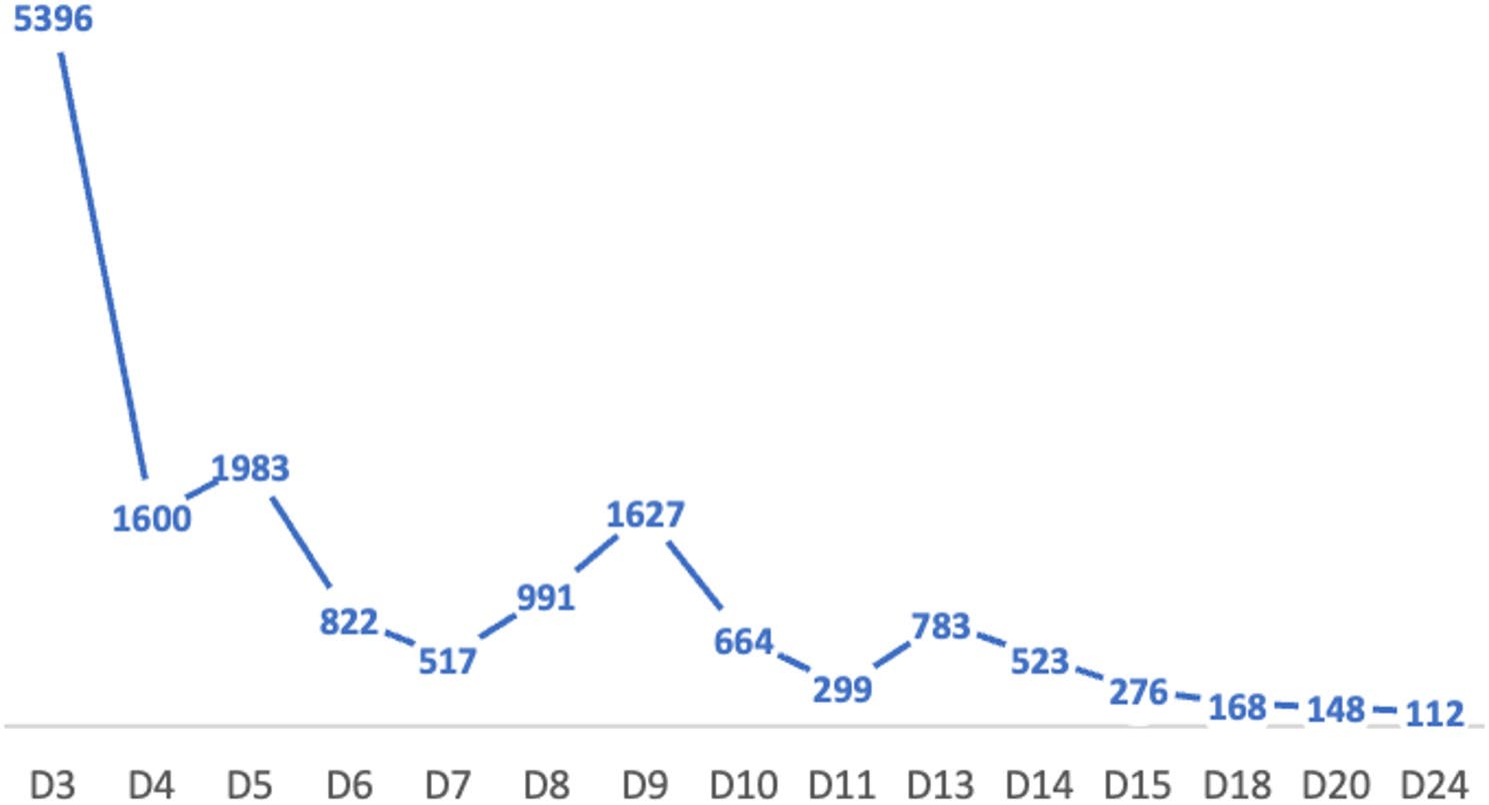
The tread of creatine kinase during our hospital

**Fig. 7:**
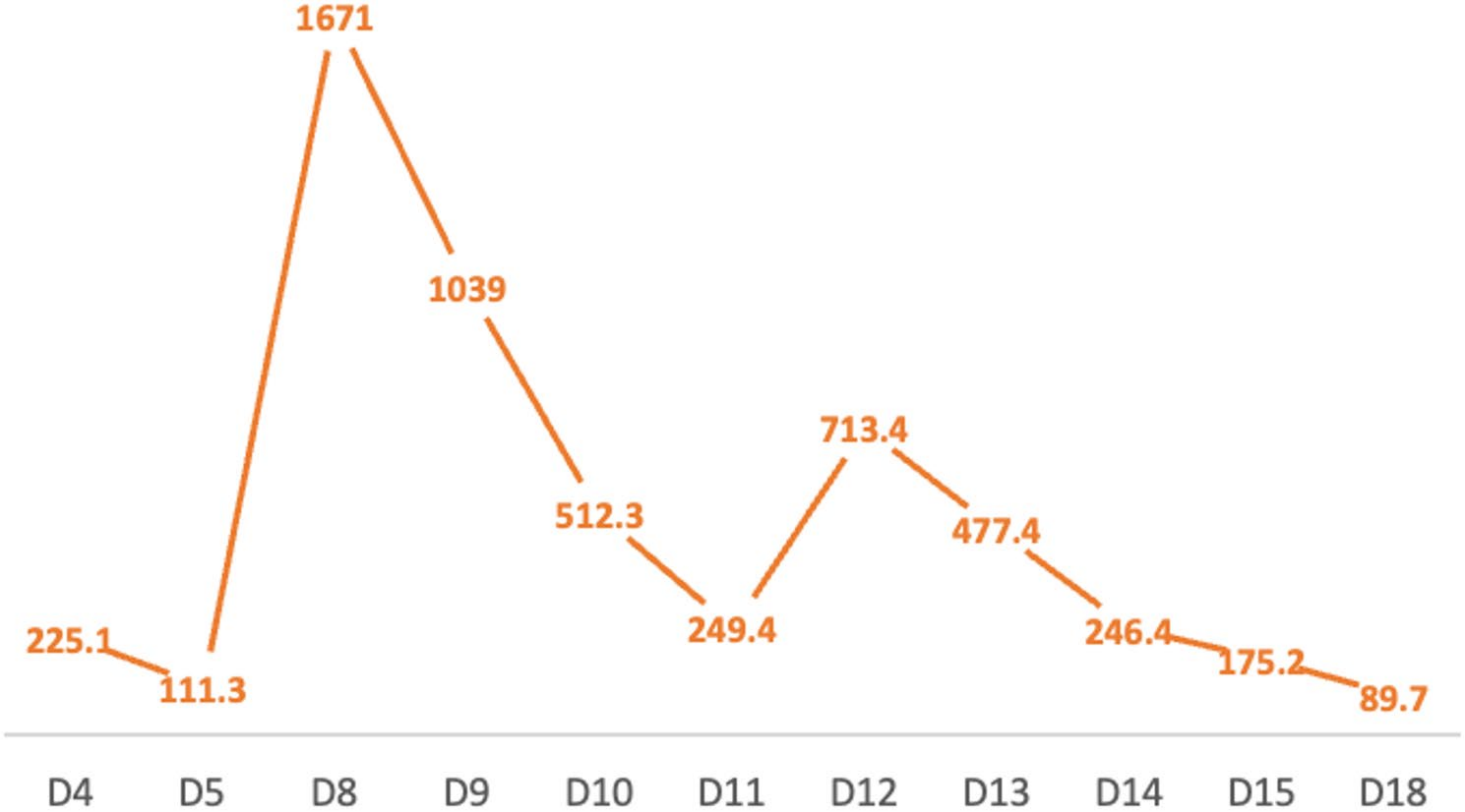
The tread of myoglobin during our hospital

The blood tests which could show the course of our patient, such as creatine kinase and myoglobin, are shown in the Figs. 6 and 7.

## Discussion

Existing literature confirms that acute diquat poisoning mainly damages the kidneys, liver, and central nervous system [5], with acute kidney injury (AKI) incidence as high as 73.3%—higher than other pesticide poisonings [6–8]. Severe cases may induce respiratory failure, heart failure, and other complications [9–10], while rhabdomyolysis is rarely reported [11, 12]. Prior to this case, only one adult case of diquat poisoning-induced compartment syndrome was documented, but the patient died of respiratory failure. Diquat can cross the placenta [13]. Shang Ruikai et al. reported another patient who ingested diquat, developed a pontine hematoma and subsequently suffered a miscarriage [14]. In 2025, the first reported case of compartment syndrome caused by diquat poisoning resulted in rescue failure, with the patient dying of respiratory failure [15]. Acute compartment syndrome (ACS), as one of the severe complications after fractures, affects 30.4% of patients with tibial shaft and proximal fractures, and is particularly common in patients with comminuted fractures or tibial plateau fractures [16–18].

In acute compartment syndrome, 69% of cases are triggered by fractures, 23% by non-fracture soft tissue trauma, and 9% by non-traumatic causes [19]. Those caused by poisoning are relatively rare. The reasons might be venomous snake bites [20], Carbon monoxide poisoning [21], and paraquat poisoning [22], rodenticide poisoning are even rarer [23–25], cocaine use [26] and snake bites [27–28]. Among 219 cases of viper bites in Switzerland, the incidence of compartment syndrome was reported to be 1.4% [26]; among 147 cases in Greece, the incidence was 1.36% [28]. However, the compartment syndrome caused by diquat poisoning haven’t been reported before.

For diquat poisoning patients with rhabdomyolysis, clinical management should include: ① basic measures: fluid replacement to expand blood volume, urine alkalinization to promote myoglobin excretion; ② renal support: renal dialysis for patients with AKI or renal failure; ③ key monitoring: close observation of limb swelling, pain, and sensory changes, with intermuscular pressure monitoring if conditions permit. Once compartment syndrome is suspected or diagnosed, emergency fasciotomy for decompression must be performed promptly, combined with incision infection prevention and early rehabilitation exercises to preserve limb function. If conditions permit, intermuscular pressure monitoring should be performed. Once compartment syndrome is diagnosed or suspected, decompressive fasciotomy of the affected limb should be performed immediately, while preventing infection at the incision site. Early rehabilitation exercises should be carried out to preserve limb function as much as possible. According to literature reports, 29.4% of rhabdomyolysis cases present with varying degrees of infiltration of CD3, CD4 and/or CD8 positive lymphocytes [29]. The most common triggers of rhabdomyolysis in adults are trauma and drugs.

The understanding of compartment syndrome following snakebite requires explicit clarification, as the underlying pathophysiology differs fundamentally from traumatic compartment syndrome. The local swelling and tissue necrosis observed in snakebite cases stem from direct venom-induced cytotoxicity, myonecrosis, and capillary leakage—not from a critical elevation of intercompartmental pressure that leads to ischemic tissue damage [27, 28]. This distinction is pivotal for guiding clinical decision-making.

Against this backdrop, antivenom therapy emerges as the mainstay of treatment. By neutralizing circulating snake venom toxins, antivenom directly addresses the root cause of tissue injury, thereby halting the progression of local swelling and necrosis [e.g., World Health Organization, 2018]. In contrast, fasciotomy (surgical compartment decompression) is not routinely recommended for snakebite-related compartment-like manifestations. Unlike ischemic compartment syndrome, where fasciotomy is life- or limb-saving, unnecessary fasciotomy in snakebite cases increases the risk of infection, bleeding, and prolonged wound healing without improving outcomes [29].

Most cases previously labeled as “snakebite compartment syndrome” are, in fact, pseudo-compartment syndromes. These are inflammatory processes driven by venom components, rather than ischemic insults. Clinical evidence confirms that such pseudo-compartment syndromes typically resolve with conservative management, including antivenom administration, local wound care, and symptomatic support (e.g., pain control, limb elevation) [28].

The clinical manifestations of acute compartment syndrome are usually described by the “five Ps”: pallor, disproportionate pain, pulselessness, paresthesia, and paralysis [30]. For patients with acute compartment syndrome, the preferred treatment is timely surgical fasciotomy. The prolongation of the time to surgical intervention is associated with an increase in tissue necrosis rate, amputation rate, and mortality. Patients diagnosed with non-traumatic compartment syndrome (FDECS) tend to have poor clinical outcomes [31].

In summary, the clinical manifestations of diquat poisoning are relatively complex, and cases complicated with rhabdomyolysis and compartment syndrome are particularly rare. The successful treatment of this case suggests that close observation, active continuous renal replacement therapy (CRRT), and timely decompressive fasciotomy are effective treatment measures.

## Data Availability

The supporting data involved in this study, including patients’ clinical examination data, laboratory test results, etc., can be provided to interested researchers on the premise of complying with ethics and privacy protection. For access to relevant data, please contact the corresponding author, Zheng Xisheng, via email: zhengxisheng@sina.com. The provision of data will strictly abide by relevant data sharing policies and regulations to ensure that patients’ privacy is not violated.
